# Psychosocial Impact of Alternative Management Policies for Low-Grade Cervical Abnormalities: Results from the TOMBOLA Randomised Controlled Trial

**DOI:** 10.1371/journal.pone.0080092

**Published:** 2013-12-30

**Authors:** Linda Sharp, Seonaidh Cotton, Julian Little, Nicola M. Gray, Margaret Cruickshank, Louise Smart, Alison Thornton, Norman Waugh, Leslie Walker, on behalf of the TOMBOLA group

**Affiliations:** 1 National Cancer Registry Ireland, Cork Airport Business Park, Kinsale Road, Cork, Ireland; 2 Obstetrics & Gynaecology, University of Aberdeen, Polwarth Building, Foresterhill, Aberdeen, Scotland; 3 Department of Epidemiology and Community Medicine, University of Ottawa, Ottawa, Ontario, Canada; 4 Centre of Academic Primary Care, University of Aberdeen, Polwarth Building, Foresterhill, Aberdeen, Scotland; 5 Department of Pathology, Aberdeen Royal Infirmary, Aberdeen, Scotland; 6 Medical School, University of Warwick, Coventry, England; 7 University of Hull, Hull, England; The University of Hong Kong, Queen Mary Hospital, Hong Kong

## Abstract

**Background:**

Large numbers of women who participate in cervical screening require follow-up for minor cytological abnormalities. Little is known about the psychological consequences of alternative management policies for these women. We compared, over 30-months, psychosocial outcomes of two policies: cytological surveillance (repeat cervical cytology tests in primary care) and a hospital-based colposcopy examination.

**Methods:**

Women attending for a routine cytology test within the UK NHS Cervical Screening Programmes were eligible to participate. 3399 women, aged 20–59 years, with low-grade abnormal cytology, were randomised to cytological surveillance (six-monthly tests; n = 1703) or initial colposcopy with biopsies and/or subsequent treatment based on colposcopic and histological findings (n = 1696). At 12, 18, 24 and 30-months post-recruitment, women completed the Hospital Anxiety and Depression Scale (HADS). A subgroup (n = 2354) completed the Impact of Event Scale (IES) six weeks after the colposcopy episode or first surveillance cytology test. Primary outcomes were percentages over the entire follow-up period of significant depression (≥8) and significant anxiety (≥11; “30-month percentages”). Secondary outcomes were point prevalences of significant depression, significant anxiety and procedure-related distress (≥9). Outcomes were compared between arms by calculating fully-adjusted odds ratios (ORs) for initial colposcopy versus cytological surveillance.

**Results:**

There was no significant difference in 30-month percentages of significant depression (OR = 0.99, 95% CI 0.80–1.21) or anxiety (OR = 0.97, 95% CI 0.81–1.16) between arms. At the six-week assessment, anxiety and distress, but not depression, were significantly less common in the initial colposcopy arm (anxiety: 7.9% vs 13.4%; OR = 0.55, 95% CI 0.38–0.81; distress: 30.6% vs 39.3%, OR = 0.67 95% CI 0.54–0.84). Neither anxiety nor depression differed between arms at subsequent time-points.

**Conclusions:**

There was no difference in the longer-term psychosocial impact of management policies based on cytological surveillance or initial colposcopy. Policy-makers, clinicians, and women themselves can be reassured that neither management policy has a significantly greater psychosocial cost.

**Trial Registration:**

Controlled-Trials.com ISRCTN 34841617

## Introduction

All screening programmes involve a balance between benefit, harm and affordability [1]. The major benefits of well-organised cervical screening are reduced incidence of, and mortality from, cervical cancer in the population [2]. However, these outcomes are achieved by a system which involves identifying large numbers of women with minor (low-grade) cytological abnormalities. In the UK, for example, around a quarter of a million cytology tests are reported as showing minor abnormalities each year [3]–[5]. Most of these women do not have cervical intraepithelial neoplasia (CIN), but are required to undergo follow-up, which may entail repeated investigations over a number of years. This follow-up is costly, both from the perspective of the health service and from the perspective of the women themselves, who may experience out-of-pocket expenses and lost time [6], [7], physical after-effects such as pain and bleeding [8]–[10] and adverse psychological effects. In terms of the last of these, the immediate negative psychological consequences of receiving an abnormal cytology result are well recognised, and include raised anxiety, cancer-related worries, body image concerns and concerns about infertility (reviewed in [11]). However, much less is known about the psychological impact of follow-up, particularly in the longer-term.

Various follow-up strategies are available [12]. Two of the main options are cytological surveillance (repeat cervical cytology tests in primary care until two or more successive tests are normal) or immediate referral for a colposcopy examination with related interventions such as biopsy and/or treatment if required [13]. The relative merits and limitations of these two approaches have been debated for several years [14]. In the large, population-based, randomised controlled trial known as TOMBOLA, nested within the UK NHS Cervical Screening Programmes (CSP), in which women were followed for three years after receipt of low-grade abnormal cytology, we found that CIN2/3 was detected earlier in women undergoing immediate colposcopy. However, the cost-effectiveness of the alternative management approaches did not differ, and immediate colposcopy was associated with higher levels of physical after-effects [15], [16]. Making informed choices between such alternatives requires a full understanding of the advantages and disadvantages of each policy but, so far, evidence on the psychological impact on women is limited; most studies have been small, considered only one management strategy and/or were not randomised. Bell et al [17] reported that the mean anxiety score among women who had been managed by surveillance for an average of 22 months was significantly higher than among those who had had colposcopy one week ago. However, the study was small (n = 150), involved women with low and high-grade cytology results, and cytological grade was confounded with management. Jones et al [18] reported higher mean recalled anxiety among 182 women with low-grade cytology who had been managed by colposcopy compared to 163 who had undergone surveillance, but the retrospective nature of the anxiety assessment, and the higher recalled anxiety reported following receipt of the initial cytology result in the colposcopy group, make the results difficult to interpret. In the only study so far to involve randomisation - of 476 women with low-grade cytology - there was no difference in mean emotional distress or anxiety scores at 12 months between women randomised to a repeat cytology test in 6-months or a choice between this and an immediate colposcopy [19]. Within the choice arm, the mean emotional distress score was higher in women undergoing surveillance, but the difference was not statistically significant.

In this paper, we use data from the TOMBOLA trial to extend this evidence-base. Our primary aim was to compare – over a 30 month period - the psychosocial impact on women of management policies based on repeat cytology tests (“cytological surveillance”) versus a colposcopic examination and, where necessary, related procedures (“initial colposcopy”). The 30-month period was intended to broadly represent the interval between screening rounds in the NHS CSPs and facilitate comparison between psychosocial and clinical results [16]. Our secondary aim was to explore whether there were differences within the 30-month window in patterns of psychosocial effects between the two management policies,

## Methods

### Participants and recruitment

**Figure 1:**
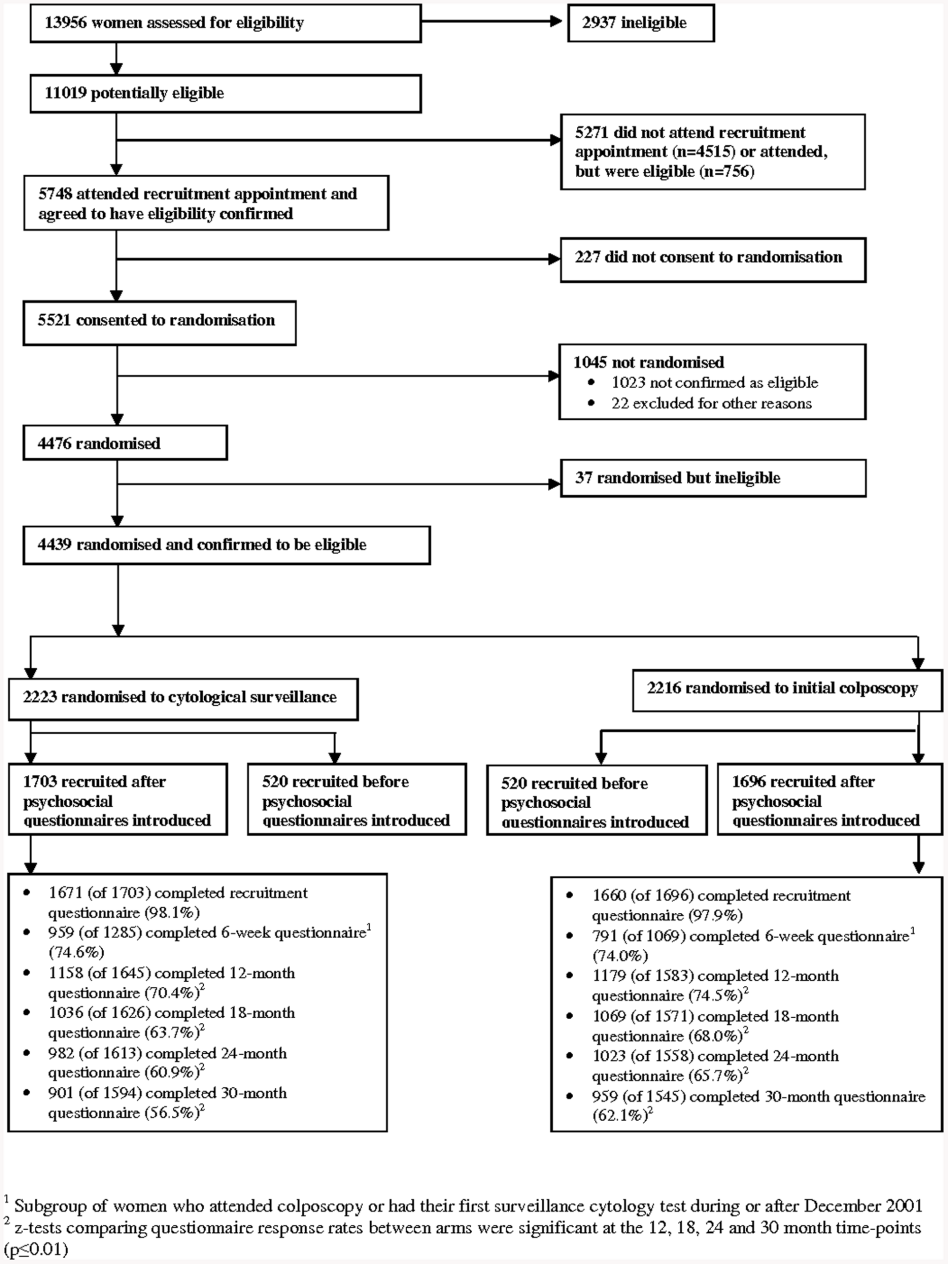
Flowchart of recruitment, randomisation and questionnaire response rates.

The protocol for this trial and supporting CONSORT checklist are available as supporting information; see Checklist S1 and Protocol S1. Full details of the design and recruitment processes are reported elsewhere [16], [20]. Recruitment is summarised in figure 1. Briefly, the trial was pragmatic (i.e. designed to inform decisions about “real-world” practice) and nested within the NHS CSPs in the UK. Eligible women were aged 20–59 with a recent routine cytology test taken October 1999–October 2002 which showed mild dyskaryosis or borderline nuclear abnormalities (BNA); broadly equivalent to low-grade squamous intraepithelial lesions (LSIL) and atypical squamous cells of undetermined significance (ASCUS) in the Bethesda system [21]. Eligible women could have up to one additional BNA result in the previous three years. Women were ineligible if they were pregnant or had had previous cervical treatment. Women attended a hospital-based recruitment clinic where recruitment was done by non-clinical trial staff. Those who consented to participate in the trial were asked to provide a cytology sample for “additional testing”. This additional testing was for high-risk human papilloma virus (HPV) infection, but neither the purpose of the sample nor the test result was revealed to women. Test results were also concealed from all of the health professionals (i.e. hospital nurses, colposcopists and clinicians and primary care practitioners) involved in the women's care. Women were subsequently randomised 50∶50 to cytological surveillance or initial colposcopy using a telephone service provided by Aberdeen University; the allocation sequence was generated centrally by the service providers and concealed from anyone directly involved with subject recruitment or delivering follow-up. The randomisation was minimised on HPV test result, age, trial centre and recruitment cytology, to ensure that the trial arms were balanced with respect to these variables as well as for the number of women in each group [22]. Women were sent a letter indicating the arm to which they had been allocated and what would happen next.

### Procedures and follow-up

The alternative management policies under evaluation were designed to mimic real-world practice in the NHS CSPs. Cytological surveillance involved repeat cytology tests every six months in primary care. Women returned to routine recall (three- or five yearly recall) if they had three consecutive normal results. Women who had a cytology test showing moderate dyskaryosis or worse, or three consecutive inadequate tests, were referred to an NHS colposcopy clinic and were managed according to local protocols. Otherwise, women remained on six-monthly tests for the duration of follow-up within the trial.

Women randomised to immediate colposcopy received an appointment to attend a hospital outpatient clinic for a colposcopy examination. Those who attended were invited to take part in a second randomisation - to immediate treatment by large loop excision of the transformation zone (LLETZ) or 2–4 targeted punch biopsies and selective recall for treatment if these showed CIN2/3 or more severe disease [23]. Women underwent colposcopy and, if the transformation zone was abnormal, they received the management to which they had been randomised. They were subsequently followed-up with six-monthly cytology tests in primary care. If the transformation zone was normal, no additional procedures were carried out at this time and women were followed by annual cytology tests in primary care. Cytological results were monitored with subsequent management (i.e. subsequent test date or referral to colposcopy) based on these. Women referred to colposcopy during follow-up attended local NHS clinics where they were treated, if required, according to the local protocol.

Women in both arms were invited to attend an exit colposcopy examination approximately three years after trial recruitment.

### Psychosocial assessments

This analysis was based on all women recruited to TOMBOLA from February 2001 onwards, the time at which the psychosocial assessments were introduced: these women were invited to complete psychosocial assessments at five time-points - at recruitment, and at four points during follow-up: 12, 18, 24 and 30 months post-recruitment. Hence, the outcomes for the analysis were self-reported by women. A sub-group of these women (all those who underwent colposcopy or had their first surveillance cytology test during or after December 2001) were invited to complete an additional assessment; this took place six weeks after either the first surveillance cytology test or completion of the colposcopy episode (i.e. colposcopy plus any biopsies or treatment, if required). The recruitment assessment was intended to provide information on potential explanatory variables and women completed the questionnaire at their recruitment clinic appointment. The six-week assessment was designed primarily to assess distress related to the procedure the women had recently undergone; subsequent assessments were designed to assess more general longer-term psychosocial effects (anxiety, depression). The six-week and subsequent questionnaires were administered by post.

The primary outcomes were significant depression and significant anxiety over the entire follow-up period. These were the percentages of women who reported significant depression or anxiety at least once over the 30-month period (i.e. at 12, 18, 24 or 30 months): henceforth this is referred to as “30-month percentage”. Significant depression and significant anxiety were assessed by the Hospital Anxiety and Depression Scale (HADS) [24], which was administered at all time-points. The HADS was originally designed to screen for clinically significant depression and anxiety in hospital medical outpatient clinics, but has been subsequently validated in primary care and community settings [25]. In addition, it has been shown to be responsive to temporal changes and discriminate well between groups with different prevalence of anxiety and depressive disorders [26], [27]. Following each assessment, we notified the GP of any woman whose HADS depression subscale score was 8 or above.

As a secondary outcome, we investigated short-term distress related to receipt of an abnormal cytology test result and its subsequent follow-up: this was the percentage of women who reported distress, as determined from the Impact of Event Scale (IES) [28], at the six-week assessment (henceforth “procedure-related distress”). The IES has been validated in a variety of contexts for the assessment of stress reactions, including intrusive experiences and avoidance of thoughts or images, after a specific traumatic event [29], [30]. In this study, women in the cytological surveillance arm were asked to complete the IES in relation to their recent surveillance cytology test; women in the colposcopy arm completed it in relation to their most recent clinic appointment, which comprised colposcopy only, colposcopy with punch biopsies or LLETZ, or a visit for treatment following CIN2/3 on punch biopsies.

The recruitment questionnaire included a section on socio-demographics and lifestyle (such as reproductive history, smoking status, and highest level of education attained). It also included the Multi-dimensional Health Locus of Control Scale (MHLCS) which measures three dimensions of health locus of control – chance, powerful others and internal [31]. This was included because locus of control was postulated to have differential effects on acceptability of clinical management.

### Statistical analysis

Questionnaire response rates at each assessment time-point were calculated based on the total number of women who remained in the trial at that time. Comparisons of trial arms were by intention-to-treat. The numbers of women who only partially completed a HADS subscale or the IES were small (between 10 and 26 at each follow-up time-point) and no imputation of missing values, or pro-rating for missing responses, was done. These women were excluded from the secondary analyses of the individual time-points, but we made the conservative decision to include them in the primary analysis of the entire 30-month window (further, we undertook a sensitivity analysis restricted to women with complete data for all time-points, described below). HADS and IES scores were strongly positively skewed and so, instead of treating these as continuous variables, for each subscale/instrument, a binary outcome variable was created based on whether or not each woman scored above a specific threshold value on that subscale/instrument. For the HADS, Zigmond and Snaith [24] provisionally suggested that scores of <8 on either scale were ‘normal’, scores of 8–10 were ‘borderline’ and scores of ≥11 were ‘abnormal’, but indicated that ideally thresholds should be validated in each setting. Bell et al [17] validated the HADS against two interview-based diagnostic scales in women in Grampian (one of the areas from which TOMBOLA participants were recruited) who had participated in cervical screening and most of whom had abnormal cytology; these authors proposed, based on analysis of receiver operator curves, that, for depression, a HADS subscale score of ≥8 provided a better cut-off than the more commonly used ≥11. A cut-off of 8 has also been recommended in guidelines for detecting depression in cancer patients [32]. This cut-off was therefore used to identify significant depression in the present study. As recommended by Zigmond and Snaith [24], a score of ≥11 was used to identify significant anxiety. Procedure-related distress at 6-weeks was defined as a total IES score of ≥9, with subcategories of mild (total score 9–25), moderate (26–43) and severe (≥44). Following common practice [33], [34], the threshold for intrusion and avoidance was a score of 20 on the relevant subscale.

To test the null hypothesis of no difference in the psychosocial impact on women (assessed in terms of significant depression and significant anxiety), over 30-months, of a policy of cytological surveillance versus a policy of initial colposcopy, we compared the 30-month percentages of significant depression and significant anxiety between the trial arms. These 30-month percentages were calculated as follows: for anxiety in the cytological surveillance arm, we divided the number of women who scored ≥11 on the HADS anxiety subscale at one or more of the 12, 18, 24 or 30-month assessments (n = 394) by the total number of women who were recruited after the psychosocial assessments were introduced and who were randomised to cytological surveillance (n = 1703; hence 30-month percentage = 23.1%).

To explore our secondary aim relating to differences in patterns of psychosocial effects between the two management policies within the 30-month window, we compared the point prevalence of significant depression, significant anxiety and procedure-related distress in each arm at all relevant individual time-points: these secondary analyses were intended mainly for descriptive purposes. Point prevalence was computed as follows: for anxiety in the cytological surveillance arm at 12-months, we divided the number of women who scored ≥11 on the HADS at the 12-month assessment (n = 218) by the number of women who completed the HADS anxiety subscale at 12-months (n = 1130; hence point prevalence = 19.3%).

Odds ratios for initial colposcopy compared to cytological surveillance were derived from logistic regression models run in STATA® (StataCorp, TX, USA) and used to test whether management arm predicted the psychological outcomes. Separate models were built for each psychosocial outcome. Our interpretation of the results was based on “fully-adjusted” odds ratios and the process for developing the fully-adjusted models was as follows. We first computed unadjusted odds ratios. Then, as recommended for trials employing minimisation [22], we adjusted odds ratios for randomisation minimisation variables (“minimally adjusted ORs”). We then further adjusted for significant confounders from among the socio-demographic, lifestyle and psychosocial variables collected at recruitment (fully-adjusted ORs). Variables considered *a priori* to be potential confounders are shown in table 1. The development of the fully-adjusted models involved an iterative process in which we took care to avoid collinearity. Confounders included in the final fully-adjusted models were significant (p<0.1) on likelihood ratio tests for the comparison of two nested models, one containing the confounder and other variables (i.e. minimisation variables and the other confounders), and one containing only the other variables. All of the final models developed though this process included HADS anxiety and/or depression at baseline (i.e. recruitment), in addition to other significant covariates. The final models had adequate fit as assessed by the Hosmer and Lemeshow test [35]. Formal statistical tests between the trial arms were conducted only for the final, fully-adjusted, models. Tests were two-sided and considered significant if p<0.05.

**Table 1:** Participants' socio-demographic, lifestyle and psychosocial characteristics at trial recruitment, full study population and subgroup invited to complete 6-week assessment.

	Full study population				Eligible for inclusion in subgroup (sensitivity) analysis			
	Cytological surveillance		Initial colposcopy		Cytological surveillance		Initial colposcopy	
	n	%	n	%	n	%	n	%
Total	1703	100	1696	100	1285	100	1069	100
**Age**								
20–29	737	43.3	727	42.9	551	42.9	456	42.7
30–39	450	26.4	456	26.9	352	27.4	286	26.8
40–49	364	21.4	357	21.0	267	20.8	228	21.3
50–59	152	8.9	156	9.2	115	8.9	99	9.3
	*chi2 (3df) = 0.21, p = 0.975*				*chi2 (3df) = 0.24, p = 0.971*			
**Eligible cytology test result**								
Mild	457	26.8	453	26.7	317	24.7	263	24.6
BNA	1246	73.2	1243	73.3	968	75.3	806	75.4
	*chi2 (1df) = 0.01, p = 0.934*				*chi2 (1df)<0.01, p = 0.970*			
**Previous BNA cytology test**								
No	1541	90.5	1542	90.9	1203	93.6	1020	95.4
Yes	162	9.5	154	9.1	82	6.4	49	4.6
	*chi2 (1df) = 0.19, p = 0.664*				*chi2 (1df) = 3.59, p = 0.058*			
**Trial centre**								
1	550	32.3	553	32.6	420	32.7	389	36.4
2	428	25.1	421	24.8	332	25.8	264	24.7
3	725	42.6	722	42.6	533	41.5	416	38.9
	*chi2 (1df) = 0.06, p = 0.972*				*chi2 (1df) = 3.58, p = 0.167*			
**High-risk HPV status**								
Negative	898	52.7	887	52.3	666	51.8	552	51.6
Positive	620	36.4	616	36.3	469	36.5	391	36.6
Missing	185	10.9	193	11.4	150	11.7	126	11.8
	*chi2 (1df) = 0.24, p = 0.889*				*chi2 (2df) = 0.01, p = 0.994*			
**Carstairs' deprivation index of area of residence**								
Least deprived (1)	252	14.8	225	13.3	184	14.3	144	13.5
2	319	18.7	314	18.5	246	19.1	205	19.2
3	260	15.3	285	16.8	193	15.0	184	17.2
4	449	26.4	451	26.6	341	26.5	289	27.0
Most deprived (5)	423	24.8	421	24.8	321	25.0	247	23.1
	*chi2 (4df) = 2.71, p = 0.608*				*chi2 (4df) = 2.96, p = 0.565*			
**Post-school education and training**								
None	452	26.7	454	26.9	337	26.4	271	25.6
Through work with qualification	338	20.0	329	19.5	256	20.1	206	19.5
Qualification other than degree	499	29.5	472	28.0	384	30.1	298	28.1
University or college degree	401	23.7	430	25.5	298	23.4	284	26.8
Missing	13	-	11	-	10	-	10	-
	*chi2 (3df) = 1.88, p = 0.597*				*chi2 (3df) = 3.80, p = 0.284*			
**Employment status**								
Full-time paid employment	850	50.2	829	49.1	647	50.6	548	51.6
Part-time paid employment	398	23.5	391	23.2	312	24.4	249	23.4
Student	146	8.6	167	9.9	102	8.0	102	9.6
Not in paid employment	300	17.7	301	17.8	217	17.0	163	15.3
Missing	9	-	8	-	7	-	7	-
	*chi2 (3df) = 1.73, p = 0.631*				*chi2 (3df) = 3.04 p = 0.386*			
**Marital status**								
Married/living as married	957	57.0	913	54.4	714	56.4	576	54.5
Divorced/widowed/separated	223	13.3	227	13.5	165	13.0	138	13.1
Single	498	29.7	538	32.1	386	30.5	343	32.5
Missing	25	-	18	-	20	-	12	-
	*chi2 (3df) = 2.62, p = 0.270*				*chi2 (3df) = 1.08, p = 0.582*			
**Ethnicity**								
White	1610	95.1	1618	96.1	1208	94.7	1022	96.4
Other	83	4.9	66	3.9	68	5.3	38	3.6
Missing	10	-	12	-	9	-	9	-
	*chi2 (1df) = 1.94, p = 0.164*				*chi2 (1df) = 4.07, p = 0.044*			
**Reproductive history**								
Never pregnant	573	34.2	558	33.3	434	34.4	357	33.8
Pregnant, no children	164	9.8	197	11.7	130	10.3	117	11.1
Pregnant with children	939	56.0	923	55.0	697	55.3	583	55.2
Missing	27	-	18	-	24	-	12	-
	*chi2 (2df) = 3.35, p = 0.187*				*chi2 (2df) = 0.38, p = 0.826*			
**Smoking status**								
Never smoked	804	47.9	804	47.8	604	47.7	507	47.9
Ex-smoker	289	17.2	287	17.1	211	16.7	187	17.7
Current Smoker	587	34.9	590	35.1	452	35.7	365	34.5
Missing	23	-	15	-	18	-	10	-
	*chi2 (2df) = 0.01, p = 0.993*				*chi2 (2df) = 0.59, p = 0.746*			
**Physical activity**								
Less than once per week	664	39.6	667	39.9	503	39.9	406	38.6
1–3 times per week	410	24.5	378	22.6	318	25.2	246	23.4
More than 3 times per week	601	35.9	627	37.5	439	34.8	399	38.0
Missing	20	-	17	-	18	-	12	-
	*chi2 (2df) = 1.85, p = 0.396*				*chi2 (2df) = 2.57, p = 0.276*			
**MHLCS chance**								
Lowest tertile (≤17)	600	38.5	601	38.6	459	39.1	383	39.2
Middle tertile (18–21)	443	28.4	453	29.1	332	28.3	281	28.8
Highest tertile (≥22)	517	33.1	501	32.2	382	32.6	313	32.0
Missing	143	-	141	-	112	-	92	-
	*chi2 (2df) = 0.36, p = 0.837*				*chi2 (2df) = 0.10, p = 0.992*			
**MHLCS internal**								
Lowest tertile (≤25)	643	40.3	641	40.4	492	41.1	400	40.1
Middle tertile (26–28)	466	29.2	494	31.1	353	29.5	300	30.1
Highest tertile (≥29)	487	30.5	453	28.5	353	29.5	298	29.9
Missing	107	-	108	-	87	-	71	-
	*chi2 (2df) = 2.03, p = 0.362*				*chi2 (2df) = 0.22, p = 0.894*			
**MHLCS powerful others**								
Lowest tertile (≤14)	558	35.5	573	36.5	424	35.8	371	37.7
Middle tertile (15–19)	526	33.4	522	33.3	394	33.3	319	32.4
Highest tertile (≥20)	490	31.1	474	30.2	366	30.9	295	29.9
Missing	129	-	127	-	101	-	84	-
	*chi2 (2df) = 0.47, p = 0.790*				*chi2 (2df) = 0.80, p = 0.671*			
**HADS anxiety at recruitment**								
Score <8	917	56.5	925	57.5	696	56.8	596	59.0
Score 8–10	334	20.6	303	18.8	250	20.4	190	18.8
Score ≥11	371	22.9	381	23.7	279	22.8	224	22.2
Missing	81	-	87	-	60	-	59	-
	*chi2 (2df) = 1.62, p = 0.444*				*chi2 (3df) = 2.14, p = 0.543*			
**HADS depression at recruitment**								
Score <8	1464	90.3	1480	92.2	1103	90.1	933	92.7
Score 8–10	111	6.8	96	6.0	88	7.2	59	5.9
Score ≥11	46	2.8	30	1.9	33	2.7	14	1.4
Missing	82	-	90	-	61	-	63	-
	*chi2 (2df) = 4.47, p = 0.107*				*chi2 (3df) = 6.35, p = 0.042*			

To assess whether randomisation resulted in balance between the trial arms in significant depression and significant anxiety at the start of follow-up, we compared the prevalence between arms at recruitment using z-tests.

### Sensitivity analyses

We conducted a sensitivity analysis of 30-month percentages which included the six-week assessment (i.e. the percentages were based on women's responses across five time-points - 6 weeks and 12, 18, 24 and 30 months). This analysis was limited to the subgroup of women who were eligible to complete the six-week assessment. To investigate whether there was any evidence of bias, the primary analyses were repeated, restricting the study population to women who had completed the psychosocial questionnaires at 12, 18, 24 and 30 months. Additional sensitivity analyses investigated the effect of changing the cut-points for the definitions of significant depression (≥11), significant anxiety (≥8) and procedure-related distress (moderate or worse ≥26).

### Ethical approval

The trial was approved by the joint research ethics committee of NHS Grampian and the University of Aberdeen, the Tayside committee on medical research ethics, and the Nottingham research ethics committee, and was registered (ISRCTN34841617). All participants provided written informed consent.

## Results

### Characteristics of participants

**Figure 2:**
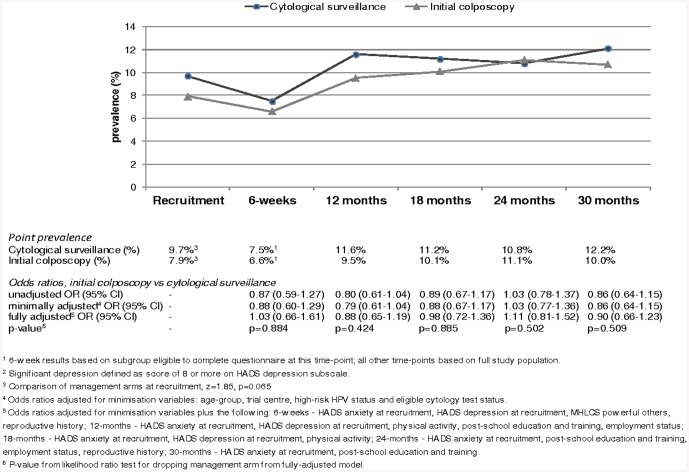
Point prevalence of significant depression, by randomisation arm, with odds ratios (OR), 95% confidence intervals and p-values (secondary analysis).

**Figure 3:**
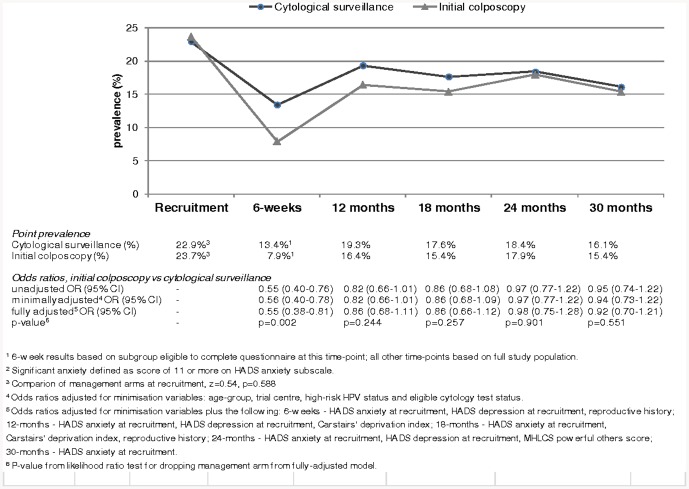
Point prevalence of significant anxiety, by randomisation arm, with odds ratios (OR), 95% confidence intervals and p-values (secondary analysis).

In total, 3399 women were eligible to take part in the psychosocial study, of whom 1703 were randomised to cytological surveillance and 1696 to initial colposcopy (figure 1). Forty-three percent of women were aged 20–29, 27% were aged 30–39, 21% were aged 40–49 and 9% were 50–59 (table 1). Just over one-quarter were recruited on the basis of a mild cytology result. Ninety-six percent of women reported their ethnic group as white. A quarter had obtained a degree from college or university. Fifty-six percent had children, 34% had never been pregnant and the remainder had been pregnant but had no children. The arms were well-balanced in terms of the randomisation minimisation variables, other socio-demographic and lifestyle factors, and locus of control. There was borderline statistically significant difference in the prevalence of significant depression at recruitment between arms (cytological surveillance 9.7%, initial colposcopy 7.9%, z = 1.85, p = 0.065; figure 2) but no difference in the prevalence of significant anxiety (figure 3).

2354 women were included in the sub-group who received the six-week assessment, 1285 in the cytological surveillance arm and 1069 in the colposcopy arm. The characteristics of these women were similar to the full study population (table 1).

### Questionnaire response rates

Ninety-eight percent of women in each arm completed the psychosocial questionnaire at recruitment (figure 1). Questionnaire response rates declined over time from 74% at six weeks to 59% at 30 months and were significantly higher in the colposcopy arm at the 12 month, and subsequent, time-points.

### Significant depression

The 30-month percentage of significant depression was 16.0% in the initial colposcopy arm compared to 16.7% in the cytological surveillance arm; this translated into a fully-adjusted odds ratio (OR) of 0.99 (95% CI 0.81–1.21; p = 0.891; table 2). The prevalence of significant depression at each time-point within the 30-month window is shown in figure 2, together with odds ratios. At all time-points, with the exception of 24 months, the point prevalence was slightly lower in the initial colposcopy arm, compared to the other arm, but these differences were not statistically significant in the fully-adjusted analyses.

**Table 2:** Numbers and 30-month percentages^1^ of women with significant depression and anxiety, by randomisation arm, with odds ratios (OR) for initial colposcopy versus cytological surveillance, 95% confidence intervals (CI) and p-values (primary analysis).

	Depression^2^	Anxiety^3^
Cytological surveillance (%; n/N)	16.7% (284/1703)	23.1% (394/1703)
Initial colposcopy (%; n/N)	16.0% (271/1696)	22.6% (383/1696)
Unadjusted OR (95% CI)	0.95 (0.79–1.14)	0.97 (0.83–1.14)
Minimally adjusted OR^4^ (95% CI)	0.95 (0.79–1.14)	0.97 (0.82–1.14)
Fully adjusted OR (95% CI)	0.99 (0.80–1.21)^5^	0.97 (0.81–1.16)^6^
p-value^7^	0.891	0.764

^1^ Percentage over entire 30-month follow-up window, based on women's responses to questionnaires at 12, 18, 24 and 30 month time points.

^2^ Significant depression defined as score of 8 or more on HADS depression subscale.

^3^ Significant anxiety defined as score of 11 or more on HADS anxiety subscale.

^4^ Odds ratio adjusted for minimisation variables: age-group, trial centre, high-risk HPV status and eligible cytology test status.

^5^ Odds ratio adjusted for minimisation variables plus HADS anxiety at recruitment, HADS depression at recruitment, Carstairs' deprivation index, post-school education and training, physical activity, reproductive history, employment status, and MHLCS powerful others score.

^6^ Odds ratio adjusted for minimisation variables plus HADS anxiety at recruitment, HADS depression at recruitment, smoking status, marital status and employment status.

^7^ P-value from likelihood ratio test for dropping management arm from fully-adjusted model.

When the analysis was repeated including the six-week time-point, the 30-month percentage was 16.7% in the initial colposcopy arm and 18.1% in the other arm, and the fully-adjusted odds ratio was not statistically significant (OR = 1.03, 95%CI 0.80–1.31, p = 0.831). The comparison of arms was unchanged when the analysis was repeated using a subscale score of ≥11 to define significant depression (not shown). There was no difference in the results when the analysis was repeated including only those women who had competed psychosocial questionnaires at all time-points (i.e. 12, 18, 24 and 30 months; not shown).

### Significant anxiety

The 30-month percentage of significant anxiety was 22.6% in the initial colposcopy arm compared to 23.1% in cytological surveillance arm. The fully-adjusted odds ratio indicated that there was no difference between the arms (OR = 0.97, 95% CI 0.81–1.16, p = 0.764; table 2). Figure 3 shows the prevalence of significant anxiety at individual time-points within the 30-month follow-up period, and associated odds ratios. At six-weeks, prevalence was lower in the initial colposcopy arm compared to the cytological surveillance arm (7.9% vs 13.4%) and the fully-adjusted odds ratio was statistically significant (OR = 0.55, 95%CI 0.38–0.81, p = 0.002). At the other follow-up time-points, the prevalence was slightly lower in the colposcopy arm, but the difference between the arms was not statistically significant. In sensitivity analyses, in which the six-week time-point was included, the 30-month percentage was slightly lower in the colposcopy arm compared to the other arm (22.7% vs 26.2%) but the fully-adjusted odds ratio was not statistically significant different from unity (OR = 0.88, 95% CI 0.71–1.09, p = 0.230). When significant anxiety was defined as a subscale score of ≥8, the results relating to the comparison of the management arms were unaffected (not shown). When the analysis was repeated including only those women who had completed psychosocial questionnaires at 12, 18, 24 and 30 months, the results were also unchanged (not shown)

### Procedure-related distress, avoidance and intrusion

At the six-week time-point, the prevalence of procedure-related distress was lower in the initial colposcopy arm compared to the cytological surveillance arm (30.6% vs 39.3%), and this difference was statistically significant in fully-adjusted analysis (OR = 0.67, 95% CI 0.54–0.84, p = 0.001; table 3). When severity of distress was considered, there was no difference in prevalence of mild or severe distress between the arms, but the prevalence of moderate distress was less than half in the colposcopy arm compared to the cytological surveillance arm (5.7% vs 13.1%). In sensitivity analyses, based on an outcome or moderate or worse distress, the fully-adjusted odds ratio was 0.49 (95% CI 0.35–0.69, p<0.001).

**Table 3:** Point prevalence of procedure-related distress, avoidance and intrusion at 6-week assessment, by randomisation arm, with odds ratios (OR) for initial colposcopy versus cytological surveillance, 95% confidence intervals (CI) and p-values (secondary analysis).

	Distress^1^	Avoidance^2^	Intrusion^3^
Cytological surveillance (%; n/N)	39.3% (345/877)	9.7% (88/904)	3.0% (27/907)
Initial colposcopy (%; n/N)	30.6% (225/736)	6.5% (49/749)	2.3% (17/755)
Unadjusted OR (95% CI)	0.68 (0.55–0.84)	0.65 (0.45–0.93)	0.75 (0.41–1.39)
Minimally adjusted OR^4^ (95% CI)	0.68 (0.55–0.84)	0.66 (0.46–0.95)	0.77 (0.41–1.43)
Fully adjusted OR^5^ (95% CI)	0.67 (0.54–0.84)	0.68 (0.46–0.99)	0.87 (0.45–1.66)
p-value^6^	0.001	0.040	0.664

^1^ Based on total IES score of 9 or more.

^2^ Based on IES avoidance subscale score of 20 or more.

^3^ Based on IES intrusion subscale score of 20 or more.

^4^ Odds ratio adjusted for minimisation variables: age-group, trial centre, high-risk HPV status and eligible cytology test status.

^5^ All odds ratios adjusted for minimisation variables plus the following: distress - HADS anxiety at recruitment; avoidance - HADS anxiety at recruitment, Carstairs' deprivation index; intrusion - HADS anxiety at recruitment.

^6^ P-value from likelihood ratio test for dropping management arm from fully-adjusted model.

The percentage of women scoring 20 or more on the avoidance sub-scale was significantly lower in the colposcopy arm, as was the fully-adjusted odds ratio (table 3). There was no difference in intrusion between arms.

## Discussion

### Longer-term impact of alternative management policies

This study investigated the psychosocial impact on women of alternative policies for the management of a low-grade abnormal cytology result over a period of 30 months. This impact was measured in terms of the proportions of women who had levels of anxiety or depression as high as someone with clinical levels, referred to here as “significant” anxiety and depression. Our observation that there was no difference in anxiety associated with cytological surveillance versus colposcopy at 12 months is consistent with the findings of Kitchener et al [19]. The larger size and longer follow-up in the current study has enabled us to extend Kitchener's findings in two ways - firstly, by showing that there is no difference in significant depression between management arms at 12 months and secondly, and more importantly, by showing that there is no difference in either outcome over the longer-term. This latter result suggest that management policies based on cytological surveillance and initial colposcopy (with related procedures where necessary) do not differ in terms of their psychosocial impact on women.

One of the concerns expressed about cytological surveillance is that persistent abnormal cytology may lead to prolonged high levels of anxiety and consequently problems with social adjustment [17], [18]. Our findings show that long-term anxiety is no more likely in women undergoing cytological surveillance than among those managed initially by colposcopy. It is worth noting, however, that although a policy of referral to colposcopy may appear to provide rapid management or resolution of the abnormal cytological result for women, in fact it is only the first step in a management process. Even women who have a normal colposcopy are likely to undergo regular cytological follow-up for some time before returning to routine recall, while – at the time of our study - women with histologically proven CIN2/3 typically underwent annual cytological and/or colposcopic follow-up for up to a decade [36]. In light of this, it is perhaps not surprising that there was no difference in the longer-term psychosocial consequences of these management policies.

### Short-term impact of alternative management policies

We have previously shown that substantial proportions of women who undergo colposcopy and related interventions following receipt of a low-grade cytology result experience screening-related distress, even when the colposcopy is normal [37]. In terms of the impact of different management options, our findings here suggest that there may be some short-term psychosocial advantage of initial colposcopy over cytological surveillance. While we found no difference in depression at six weeks, we did observe lower levels of anxiety and procedure-related distress (and avoidance in particular) in the colposcopy arm. That the symptoms of anxiety and distress overlap quite considerably might account for the similarity in findings for these two constructs. Indeed, in our population, distress and anxiety were strongly related: 21% of women who scored in the range for distress also had significant anxiety, compared to 4% of those who were not distressed.

Distress and anxiety are common reactions to uncertainty [38]. It is highly plausible that undergoing follow-up for an abnormal cytology result would cause uncertainty, fear of future events, and feelings of lack of control – and that these, in turn, could generate anxiety and distress. The higher level of avoidance in the surveillance arm suggests that this may be what women use to cope with the increased distress and anxiety associated with that management option. The lower odds of anxiety and distress in the colposcopy arm could be explained by the fact that women in that arm had attended hospital for investigation, and possibly treatment, and had received what they perceived as a “definitive result” (irrespective of what that result was), thus resolving – to some extent - the uncertainty. Rapid resolution of anxiety and relief that “disease” has been treated has previously been suggested to be one the main advantages of colposcopy over cytological surveillance [17], [18]. The fact that the difference in anxiety between arms in the current study was not sustained in the longer-term demonstrates that any benefit of colposcopy in terms of providing women with reassurance is transient.

Various factors may complicate the interpretation of the six-week comparison between arms. The six-week assessment was conducted in a sub-group of the study population, and women who did not attend for colposcopy or a first surveillance smear were not included. Default from colposcopy was higher than for cytological surveillance (6.7% vs 2.4%) [39], [40]. If raised anxiety was associated with default, as has been previously suggested [41], this would mean that we would have tended to underestimate the prevalence of anxiety to a greater extent in the colposcopy, than in the cytology, arm. However, since the level of default was relatively low, it would seem unlikely that this could entirely explain the observed difference between the arms. On the other hand, it should also be noted that, at the time of the assessment, women in the cytological surveillance arm were chronologically further from recruitment (and their abnormal cytology test) than women in the other arm. If women's emotional reactions to abnormal cytology decline over time (as might have been expected *a priori*), this could have served to bias the odds ratios towards the null. Finally, we chose to administer the questionnaire at six-weeks because we anticipated that women would have received results of their cytology test or colposcopy and any related procedures before receiving the questionnaire. Colposcopy and histology results were reported to women by the TOMBOLA trial office and the turnaround time was generally two weeks or less. By contrast, cytology results were reported directly from laboratories. We know that at some times during the trial there were significant delays in cytology reporting, and it is therefore possible that some women may not have received their result before completing the questionnaire. This could have artificially inflated the prevalence of anxiety at six-weeks in the cytological surveillance arm.

### Patterns of anxiety and depression during follow-up

Although the statistical methodology was not optimal for the detection of temporal changes, one of the striking findings from the secondary analysis related to the pattern of anxiety during the follow-up period. The heightened level of anxiety at recruitment (23% of women scored in the range for significant anxiety eight weeks, on average, after they received their abnormal cytology test result) has previously been described by ourselves and others [42]–[45]. By 30 months later, prevalence had fallen by almost one-third, but the pattern between these points was distinctive: at the six-week assessment only around 10% scored in the range for significant anxiety and by 12-months this had risen to 17/18% and remained around that level thereafter. Although our study did not include a matched external comparison group, estimates of the prevalence of anxiety (using the same definition as ours) in other unselected series provide some indication of likely levels in the general population; these range from 10% among 2048 women aged 18–65 in the Netherlands, to 12% among 604 women aged 30–75 in Denmark and 15% among 978 women aged 18–91 in the UK [46]–[48]. Our results, therefore, suggest that an initial management intervention (either colposcopy or cytology test) leads to some resolution of the anxiety generated by initial receipt of a low-grade cytology result, but that there is a degree of persistent heightened anxiety in the longer-term.

The pattern of depression during follow-up was quite different to that for anxiety. Around 8.5% of women scored in the range for significant depression shortly after receipt of the abnormal cytology result; there was a modest dip, to 7%, at six-weeks followed by a modest rise to 10% at 12-months, remaining at that level thereafter. The prevalence of depression in the current study was lower than among women who were undergoing cytology surveillance (16%) or had recently had colposcopy (24%) in the study of Bell et al [17], but that study included women with high-grade and low-grade abnormal cytology results. Not surprisingly, prevalence in the current study was lower than among women in Grampian who had been treated for gynaecological cancer (16%) [49]. It was also lower than that reported in an unselected series of 978 UK women aged 18–91 (13%) [48], and of a similar level to that among 75 women in Grampian who had had a normal cytology test in the previous year (7%) [17], which could be explained by lower levels of participation in cervical screening by women who are depressed [50], [51]. Overall these comparisons suggest that receipt of a low-grade cytology result and subsequent follow-up is not associated with significant depression. This is reassuring but perhaps unsurprising since depression would not be a typical first reaction to medium length stressors, such as management of an abnormal cytology result.

### Strengths & limitations

This large, population-based, trial was nested within the NHS CSPs. The overall participation rate in the trial was 52%. Two strands of evidence suggest that the generalisability of the results is likely to be high. Firstly, the ratio of BNA to mild cytology results among trial recruits was similar to that observed in the CSPs [16]. Secondly, the cumulative incidence of CIN2/3 during follow-up was similar to that observed in other studies conducted within the screening programme [52], [53]. Moreover, TOMBOLA was a pragmatic trial and the management policies evaluated mimicked those offered by the CSPs. Other than the recruitment HPV test, women received no interventions during follow-up which were not part of standard care. As regards the HPV test, women were not informed that they were being tested specifically for HPV, nor was the result revealed to them or their doctors. Although we have previously described associations between HPV status and anxiety [54], the comparison between the management arms reported here would be expected to be unbiased.

Trial participation was lower among younger women, and we have previously shown that the prevalence of anxiety is higher in this group [42]. In addition, women who were anxious or depressed at recruitment were less likely to complete subsequent questionnaires, and anxiety or depression at recruitment were strong predictors of these outcomes at subsequent time-points. These two issues mean that we are likely to have underestimated the overall prevalence of negative psychosocial effects in women with low-grade cytological abnormalities. Questionnaire response rates at the 12-month assessment, and later time-points, were lower in the cytological surveillance arm. Therefore, the underestimation may be slightly more pronounced in this arm. This is unlikely, however, to have been of a sufficient magnitude to conceal a true significant difference in the psychosocial impact of the management policies. We do acknowledge that we analysed multiple outcomes across multiple time points and conducted extensive sensitivity analyses. We took the decision a priori not to adopt an adjust significance level because the most commonly used method, the Bonferroni correction, is known to be conservative. Instead we took the approach of clearly stating our primary hypothesis and hence primary analysis (i.e. comparison between trial arms of 30-month percentages of significant depression and significant anxiety), considering all other comparisons secondary or exploratory. However, this does mean that multiple testing is a consideration and some care is needed in interpretation of the marginal p-values.

This is the first study to have prospectively assessed the psychosocial impact of low-grade cytology tests and their subsequent management beyond 12 months. The instruments that we used have been extensively validated. There was little *a priori* evidence on which to base selection of the time-points at which to conduct the psychosocial assessments; we therefore selected these to avoid being too close to expected follow-up visits, and thus avoid the spikes of anxiety which may be associated with these. Other than the short information leaflets which we provided as part of the trial, we had no control over the information women received (or sourced for themselves) about the management policies or their results. Whilst we would expect that there would be substantial differences between individual women in terms of information seeking and receipt, the randomisation should have provided balance between the arms in this regard.

### Conclusions

In this large, population-based, trial we found no difference in the psychosocial impact of management policies based on cytological surveillance or initial colposcopy over 30-months. Policy-makers, clinicians, and women themselves can be reassured that neither management policy has a significantly greater psychosocial cost.

## Supporting Information

Checklist S1 **CONSORT Checklist - The Trial Of Management of Borderline and Other Low-grade Abnormal smears (TOMBOLA).**
(DOC)

Protocol S1 **Protocol for the Trial Of Management of Borderline and Other Low-grade Abnormal smears (TOMBOLA).**
(PDF)
