# Supplementary material for: Psychosocial Impact of Alternative Management Policies for Low-Grade Cervical Abnormalities: Results from the TOMBOLA Randomised Controlled Trial
Source: PLoS One. 2013 Dec 30;8(12):e80092. doi: 10.1371/journal.pone.0080092 (PMC3875419; doi:10.1371/journal.pone.0080092)
Supplement: Checklist S1 — CONSORT Checklist - The Trial Of Management of Borderline and Other Low-grade Abnormal smears (TOMBOLA). (DOC) [file pone.0080092.s001.doc]

**Supporting Information S1: CONSORT Checklist - The Trial Of Management of Borderline and Other Low-grade Abnormal smears (TOMBOLA)**

| PAPER SECTION And topic | Item | Description | Reported on  Page # |
| --- | --- | --- | --- |
| *TITLE & ABSTRACT* | 1 | [How participants were allocated to interventions](http://www.consort-statement.org/examples1.htm) (*e.g*., "random allocation", "randomized", or "randomly assigned"). | Included in *Title* and *Abstract* |
| *INTRODUCTION* Background | 2 | [Scientific background and explanation of rationale.](http://www.consort-statement.org/examples2.htm) | *Introduction* |
| *METHODS* Participants | 3 | [Eligibility criteria for participants](http://www.consort-statement.org/examples3a.htm) and the [settings and locations where the data were collected](http://www.consort-statement.org/examples3b.htm). | Included in *Methods: participants and recruitment*. References also provided to paper describing trial protocol (Cotton et al., 2006: Pubmed ID: 16765101) and paper describing the clinical outcomes associated with the two interventions (TOMBOLA Group, BMJ, 2009: Pubmed ID:19638646) |
| Interventions | 4 | [Precise details of the interventions intended for each group and how and when they were actually administered.](http://www.consort-statement.org/examples4.htm) | Included in *Methods: procedures and follow-up* |
| Objectives | 5 | [Specific objectives and hypotheses](http://www.consort-statement.org/examples5.htm). | *Abstract* and final sentence of *Introduction* |
| Outcomes | 6 | [Clearly defined primary and secondary outcome measures](http://www.consort-statement.org/examples6a.htm) and, when applicable, any [methods used to enhance the quality of measurements](http://www.consort-statement.org/examples6b.htm) (*e.g.*, multiple observations, training of assessors). | Included in *Methods*: *psychosocial assessments* and *Methods:* *statistical analysis* |
| Sample size | 7 | [How sample size was determined](http://www.consort-statement.org/examples7a.htm) and, when applicable, [explanation of any interim analyses and stopping rules](http://www.consort-statement.org/examples7b.htm). | This study relates to a subsample of women included in a larger RCT. The subjects were women recruited to the trial after the date at which the psychosocial assessments were implemented. The trial was powered for the detection of differences in the main clinical outcome, CIN 2/3 or more severe disease. Details of the sample size calculations are provided in the paper describing the clinical results (TOMBOLA Group, BMJ, BMJ, 2009. Pubmed ID: 19638646). |
| Randomization -- Sequence generation | 8 | [Method used to generate the random allocation sequence, including details of any restrictions](http://www.consort-statement.org/examples8a.htm) (*e.g*., blocking, stratification) | Details of randomisation provided in *Methods: participants and recruitment*. Randomisation was stratified by various factors and this is described in this section. |
| Randomization -- Allocation concealment | 9 | [Method used to implement the random allocation sequence](http://www.consort-statement.org/examples9.htm) (*e.g*., numbered containers or central telephone), clarifying whether the sequence was concealed until interventions were assigned. | A central telephone service was used (see *Methods: participants and recruitment)* |
| Randomization -- Implementation | 10 | [Who generated the allocation sequence, who enrolled participants, and who assigned participants to their groups.](http://www.consort-statement.org/examples10.htm) | Described in M*ethods:participants and recruitment*. The allocation sequence was generated centrally and unknown to anyone directly involved in the trial. |
| Blinding (masking) | 11 | [Whether or not participants, those administering the interventions, and those assessing the outcomes were blinded to group assignment.](http://www.consort-statement.org/examples11a.htm) If done, [how the success of blinding was evaluated](http://www.consort-statement.org/examples11b.htm). | See *Methods: procedures and follow-up* and the first line of *Methods: psychosocial assessments*. Once the allocation had been done, women were informed of the group to which they had been randomized. Women allocated to cytological surveillance attending for repeat smears in primary care so their GP and practice nurse would have been aware of the group to which they had been assigned. Women allocated to colposcopy attended a hospital clinic where nurses, colposcopists and gynecologists would have been aware of the policy to which they were randomized. Outcomes were assessed by women themselves: no clinical staff were involved in assessment of outcomes. |
| Statistical methods | 12 | [Statistical methods used to compare groups for primary outcome(s)](http://www.consort-statement.org/examples12a.htm); [Methods for additional analyses,](http://www.consort-statement.org/examples12b.htm) such as subgroup analyses and adjusted analyses. | See *Methods: statistical analysis* |
| RESULTS  Participant flow | 13 | [Flow of participants through each stage](http://www.consort-statement.org/examples13a.htm) (a diagram is strongly recommended). Specifically, for each group report the numbers of participants randomly assigned, receiving intended treatment, completing the study protocol, and analyzed for the primary outcome. [Describe protocol deviations from study as planned, together with reasons.](http://www.consort-statement.org/examples13b.htm) | Full details provided in *figure 1* |
| Recruitment | 14 | [Dates defining the periods of recruitment and follow-up.](http://www.consort-statement.org/examples14.htm) | See *Methods:participants and recruitment* and final sentence of M*ethods: procedures and follow-up* |
| Baseline data | 15 | [Baseline demographic and clinical characteristics of each group.](http://www.consort-statement.org/examples15.htm) | See *Results: characteristics of participants* and *table 1.* |
| Numbers analyzed | 16 | [Number of participants (denominator) in each group included in each analysis and whether the analysis was by "intention-to-treat"](http://www.consort-statement.org/examples16.htm). State the results in absolute numbers when feasible (*e.g*., 10/20, not 50%). | All comparisons of trial arms were by intention-to-treat (see *Methods:statistical analysis*). The number of women in each arm is shown in *table 1*. The number of women who completed questionnaire at each time point is shown in *figure 1.* |
| Outcomes and estimation | 17 | [For each primary and secondary outcome, a summary of results for each group, and the estimated effect size and its precision](http://www.consort-statement.org/examples17.htm) (*e.g.*, 95% confidence interval). | See *figures 2* (depression) and *3* (anxiety) and *tables 2* and *3*. |
| Ancillary analyses | 18 | [Address multiplicity by reporting any other analyses performed](http://www.consort-statement.org/examples18.htm), including subgroup analyses and adjusted analyses, indicating those pre-specified and those exploratory. | Unadjusted, minimally adjusted and fully-adjusted odds ratios are given in *tables 2 and 3* and in *figures 2 and 3*. A subgroup analysis was done restricting to women who were eligible to complete the 6-week assessment; these results are described in *table 2*. All sensitivity analyses conducted are described in *Methods: statistical analysis* and in the *Results* |
| Adverse events | 19 | [All important adverse events or side effects in each intervention group.](http://www.consort-statement.org/examples19.htm) | There were no adverse events. |
| DISCUSSION Interpretation | 20 | [Interpretation of the results](http://www.consort-statement.org/examples20.htm), taking into account study hypotheses, sources of potential bias or imprecision and the dangers associated with multiplicity of analyses and outcomes. | Strengths and limitation of the study are specifically addressed in the *Discussion*. Results are interpreted in the context of other available evidence (*Discussion*) |
| Generalizability | 21 | [Generalizability (external validity) of the trial findings.](http://www.consort-statement.org/examples21.htm) | Addressed in *Discussion:strengths and limitations*, first paragraph |
| Overall evidence | 22 | [General interpretation of the results in the context of current evidence.](http://www.consort-statement.org/examples22.htm) | Addressed in the *Discussion* |
